# Annotations

**Published:** 1903-10-31

**Authors:** 


### Annotations.

### Working Men and Hospital Management.

There were some unusually interesting proceedings at the adjourned half-yearly meeting of the Governors of the Merthyr General Hospital which was held the other day. The object of the meeting was to receive a report from the workmen of the district relative to the maintenance of the new additions to the hospital which are now in course of construction. In the discussion which arose on the report, several of the representatives of the men employed in the local works and collieries, who subscribe to the institution and have been asked to augment their contributions in order to meet the additional expense involved in the extension, took part. It was intimated by one of these speakers that the workmen he represented "had decided not to increase their present contributions until the matter of an increase in the medical staff had been settled," and he added that "they thought that every person who met with an accident in the works or collieries should have the right to be attended in the hospital by his own medical man." Several others, however, representing a similar class, did not go so far as this, but declared that they would be quite satisfied with an increase in the medical staff, which they regarded as being too small. A miners' agent, representing the Dowlais colliers, announced that they were quite prepared to pay a halfpenny per man per week if the doctor question were properly settled, but otherwise they could not think of doing so. Dr. Biddle, while affirming that the suggestion to bring in all the medical men in the district was impracticable, observed that if the workmen came to a unanimous conclusion to increase their contributions, subject to the medical staff being simply enlarged, he would heartily support them, and it was ultimately arranged that the men should meet again and formulate in writing exactly what they wanted, as there was now so much diversity of opinion. We agree with the line taken by Dr. Biddle, but the notable and most satisfactory feature of the affair is that the colliers should have manifested in such an emphatic manner the interest they feel in the management of the Merthyr Hospital. Like other institutions, hospitals suffer more from indifference than from anything else, and it is a healthy and encouraging sign when a large and representative body of working men, instead of adopting the "don't care" attitude, not only give their money to a charity but also concern themselves as to how it going to be spent.

### The Dose of Carbolic Acid.

The earlier stages in the movement by which a number of diseases have been proved to be due to the presence in the body of micro-organisms, excited, and naturally so the expectation of an active development of successful therapeutics. It seemed an eminently reasonable conclusion that as the nature of the various pathogenic bacteria became known means would surely be devised to control or neutralise their activities. So far as laboratory experiments are concerned this anticipation was very largely realised. Unfortunately the agents which in such experiments were most successful in destroying the various kinds of specific micro-organisms were exactly those having a poisonous action on the living tissues. Hence it has for the most part been deemed impossible to introduce into the body anti-bacterial substances in doses sufficiently large to destroy the germs of specific diseases, and, with the large exception of anti-toxic serums, the discoveries of the bacteriologists have not been attended by any corresponding therapeutic development. Some results recently obtained in the treatment of bubonic plague by large doses of carbolic acid suggest the possibility that this partial failure may be due in greater or less measure to the use of insufficient doses. It has been found by Dr. J. C. Thomson that patients suffering from plague can take carbolic acid in amounts far in excess of the orthodox practice and without inconvenience or harm. In a number of instances he has prescribed a dose of 12 grains repeated every two hours, and has continued the administration for several days. Carboluria and some digestive disturbance were only exceptional occurrences and were in no instance the cause of anxiety. Whatever may be the exact explanation of these facts Dr. Thomson's experience certainly emphasises the necessity for therapeutic courage. Of the dangers of excessive doses we hear much. But there is equally a possibility of loss from error in the opposite direction.

### Dirty Money and Disease.

It is told of an English lady who went to live in Scotland that she said, when she received the very grimy one pound notes which are so popular in that country, and which pass through so many hands before being called in, that "never before had she understood what was meant in the Bible by 'filthy lucre.'" The sentiment will be echoed by all who have to deal with a paper currency for small sums. But it is sometimes forgotten that the paper notes have the advantage of showing the dirt, which is as present, though not as obvious, on our coins. A medical writer in a contemporary mentions that he saw a man who was clearly suffering from an infectious skin disease of the hands paying a tramway fare without a thought of the ill he might convey with the coins he passed to the conductor. The conductor, when warned, was effusively grateful for the warning, and promised, for his own protection, to wear gloves in the future. But there was no protection thought of for the people who might next handle these dirty coins. To trace infection of any kind to a particular coin may be impossible, but one may still realise that infection may so be brought among us. Thackeray speaks of it having been once the custom at a club to bring a member the change that he needed in "washed silver." The novelist works this out into an apologue, to indicate that in a gentleman a certain cleanliness of life and thought as well as of habit, is expected, and indeed, one could moralise *ad libitum* on the theme. It certainly does not follow that infection lurks in every penny, the previous travels and antecedents of which we have not investigated, but the incident may serve as a reminder that money may advisedly be handled with some little caution, seeing that we do not know through whose hands it has passed.

